# Fabrication of Taper Free Micro-Holes Utilizing a Combined Rotating Helical Electrode and Short Voltage Pulse by ECM

**DOI:** 10.3390/mi10010028

**Published:** 2019-01-03

**Authors:** Yong Liu, Minghong Li, Jingran Niu, Shizhou Lu, Yong Jiang

**Affiliations:** Associated Engineering Research Center of Mechanics and Mechatronic Equipment, Shandong University, Weihai 264209, China; rzliuyong@sdu.edu.cn (Y.L.); 201614777@mail.sdu.edu.cn (M.L.); 201614780@mail.sdu.edu.cn (J.N.); 201736323@mail.sdu.edu.cn (Y.J.)

**Keywords:** ultra-short pulse, helical electrode, theoretical model, electrochemical drilling (ECD), micro holes

## Abstract

Fabrication of the injection nozzle micro-hole on the aero engine is a difficult problem in today’s manufacturing industry. In addition to the size requirements, the nozzle micro-hole also requires no burr, no taper and no heat-affected zone. To solve the above problem, an ultra-short voltage pulse and a high-speed rotating helical electrode were used in electrochemical drilling (ECD) process. Firstly, a theoretical model of ECD with ultra-short voltage pulse was established to investigate the effects of many predominant parameters on machining accuracy, and the effect of rotating helical electrode on the gap flow field was analyzed. Secondly, sets of experiments were carried out to investigate the effects of many key parameters on machining accuracy and efficiency. Finally, the optimized parameters were applied to machine micro holes on 500 μm thickness of GH4169 plate, and micro-holes with the diameter of 186 μm with no taper were machined at the feed rate of 1.2 μm/s. It is proved that the proposed ECD process for fabricating micro-holes with no taper has a huge potential and broad application prospects.

## 1. Introduction

With the development of micro electro-mechanical system (MEMS), the demand for micro components is increasing [1]. As common component structures, the quality of micro holes is determined by the machining accuracy, taper of holes and the machining efficiency. How to machine high quality micro holes efficiently is a research hotspot for scholars from many countries.

Thus far, there are many common technologies to machine micro holes, such as mechanical drilling, lasers, punching, electrical discharging machining (EDM), etc., but they all have some drawbacks such as machining stress, tool wear, and taper problems [1,2,3,4]. Electrochemical machining, as one of the most promising machining technologies, can be used to machine micro holes [2]. To improve the machining accuracy of electrochemical machining, Rolf Schuster proposed using ultra-short voltage pulses to localize the machining region in the process of electrochemical micromachining, and some 3D micro structures were machined on copper sheet [5]. Then, many researchers applied ultra-short voltage pulses to improve the machining accuracy of micro holes [6,7,8]. According to experiments, utilizing side-insulated electrode in the process of ECD can also improve the machining accuracy of holes [9,10]. Side-insulated electrodes are used to avoid the phenomenon of secondary electrolysis, thus decreasing the taper of holes obviously [10,11,12]. Fang et al. applied a potential difference between an auxiliary electrode and the anode to improve the accuracy of exit holes [13]. To decrease the taper of holes, Kim et al. used a disk-type electrode to machine micro structures [14]. Liu et al. fabricated micro spherical electrode, and drilled micro holes without taper by applying the electrodes [15]. Then, to improve the machining efficiency, multiple electrodes are applied to machining micro holes simultaneously, increasing the efficiency obviously [14,16,17]. Taking advantage of single rotary helical electrodes in the process of ECD can enhance the maximum feed rate, and the maximum feed rate can be improved by increasing rotating speed [18,19,20,21], thus the machining efficiency can be improved by using rotary helical electrodes. Wu et al. developed a low-cost desktop micro electro-chemical machining (ECM) and fabricated micro-holes in the tungsten cemented carbide (WC-Co) workpiece with spindle electrode [22]. In addition, to machine micro-holes on silicon materials, various electrochemical composite machining methods have been studied [23,24,25,26,27,28].

In this work, ultra-short voltage pulse and high-speed helical electrode were combined in ECD process to improve the machining accuracy, decrease the taper of holes, and improve the machining efficiency. The theoretical model was established and experiments were carried out to investigate the influence of many predominant parameters on the machining drilling process. Finally, high-accuracy micro array holes were fabricated efficiently applying the optimized parameters.

## 2. Experimental

### 2.1. Experimental System

**Figure 1:**
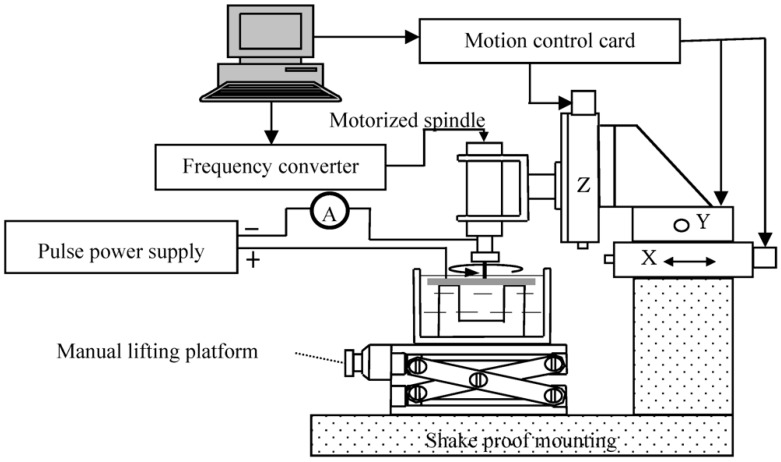
Schematic diagram of experimental system.

To perform the ECM experiments presented below, an experimental system with high-precision was constructed. The experimental system consists of various sub-components, e.g., electrodes unit, pulse power supply system, servo-control feed unit, etc. Figure 1 presents a schematic view of the various system components of the experimental system.

The electrodes unit consists of the motorized spindle, frequency converter, tool holder, helical electrode, workpiece, etc. The helical electrode can be rotated driven by the motorized spindle, whose rotating speed is controlled by the frequency converter. The maximum rotating speed of the motorized spindle is 40,000 r/min, which meets the experimental requirements. The pulse power supply system is made up with ultra-short pulse power supply, mechanical ammeter, electricity-conductive device, etc. The mechanical ammeter was used to find the suitable machining position and monitor the machining current.

### 2.2. Theoretical Model of ECD with Ultra-Short Voltage Pulse

**Figure 2:**
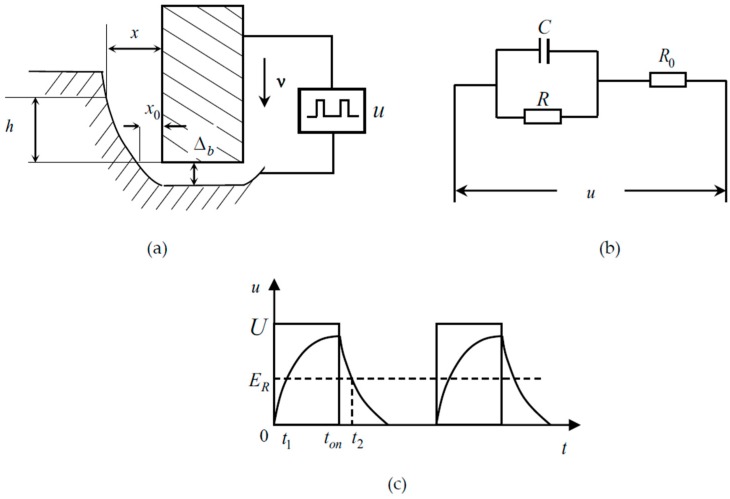
Process and equivalent circuit of electrochemical drilling (ECD) by ultra-short voltage pulse: (**a**) schematic diagram; (**b**) equivalent circuit diagram; and (**c**) waveform of the reaction voltage.

The principle and equivalent circuit of ECD with the ultra-short voltage pulse is shown in Figure 2 [29]. In Figure 2a, the cylindrical electrode feeds down at the rate of ν, the machining gap is $\Delta_{b}$, the initial side gap is $x_{0}$, and the side gap at the position of h from the bottom of the electrode is x. Due to the existence of double layer capacitor, the charging and discharging applies the ultra-short voltage pulse to ECD. Figure 2b shows the equivalent circuit of ECD. The capacitance between the cathode and anode is *C*, the electrochemical reaction resistance is R, and the sum of the electrolyte resistance and the internal resistance of the power source is $R_{0}$.

According to the transient response analysis of the circuit [5,14], the reaction voltage between cathode and anode is $u_{1}$. By $B=\frac{R}{R+R_{0}}$, $\tau =\frac{RR_{0}}{R+R_{0}}C$, the voltage can be expressed in every cycle as

(1) $$u_{1}=\{\begin{array}{cc} uB(1-e^{-\frac{t}{\tau }}) & \left(0\leq t<t_{on}\right)\\ uB(1-e^{-\frac{t_{on}}{\tau }})e^{{}^{-\frac{t}{\tau }}} & \left(t_{on}\leq t<t_{on}+t_{off}\right) \end{array}$$

**Figure 3:**
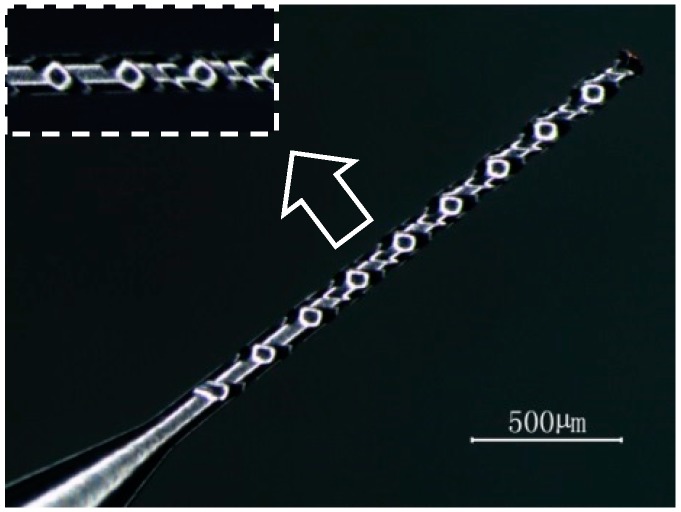
Structure of helical electrode.

The waveform of the reaction voltage $u_{1}$ is shown in Figure 2c, and the decomposition voltage of the material is $E_{R}$. According to electrochemical theory, only when the reaction voltage is higher than the decomposition voltage will the workpiece be machined. The line of $E_{R}$ in Figure 3 has the point of intersection at $t_{1}$ and $t_{2}$ with the reaction voltage waveform, which means the workpiece can be machined from $t_{1}$ to $t_{2}$ in every cycle. According to Figure 2c, $t_{1}$ and $t_{2}$ can be expressed as

(2) $$\{\begin{array}{c} t_{1}=-\tau \ln(1-\frac{E_{R}}{uB})\\ t_{2}=t_{on}-\tau \ln(\frac{E_{R}}{uB})+\tau \ln(1-e^{-\frac{t_{on}}{\tau }}) \end{array}$$

In the process of ECD, the average machining gap is expressed as $\bar{\Delta_{b}}$, and the corrosion rate $\nu_{a}$ of workpiece can be represented as [20]

(3) $$\nu_{a}=\frac{\eta \omega \sigma_{di}(u_{1}-E_{R})}{\bar{\Delta_{b}}}$$

where $\sigma_{di}$ is the conductivity of electrolyte in the machining gap. To maintain the invariance of the average machining gap, the depths of etching and feeding down should be equal in every cycle.

(4) $$\int_{t_{1}}^{t_{2}}\frac{\eta \omega \sigma_{di}(u_{1}-E_{R})}{\bar{\Delta_{b}}}\mathrm{dt}=\nu T$$

Then, the average machining gap $\bar{\Delta_{b}}$ can be expressed as

(5) $$\bar{\Delta_{b}}=\frac{\eta \omega \sigma_{di}(uB-E_{R})}{\nu T}t_{on}+\frac{\eta \omega \sigma_{di}\tau }{\nu T}\left[\left(uB-E_{R})\ln(1-\frac{E_{R}}{uB})+E_{R}\ln\frac{E_{R}}{uB}-E_{R}\ln(1-e^{-\frac{t_{on}}{\tau }}\right)\right]$$

Due to small radius of curvature at the bottom of the electrode, the initial value of the side gap $x_{0}$ is equal to the average machining gap $\bar{\Delta_{b}}$.

Similarly, the corrosion rate $\nu_{n}$ in the side direction could be expressed as

(6) $$\nu_{n}=\frac{\eta \omega \sigma_{ce}(u_{1}-E_{R})}{x_{n}}$$

where $x_{n}$ is the side gap at the position of and $\sigma_{ce}$ is the conductivity of the electrolyte in the side gap. Thus, the increment of the side gap $\Delta x_{n}$ is generated during one cycle, which can be expressed as

(7) $$\Delta x_{n}=\int_{t_{1}}^{t_{2}}\frac{\eta \omega \sigma_{ce}(u_{1}-E_{R})}{x_{n}}\mathrm{dt}$$

Then, the increment of the side gap $\Delta x_{n}$ can be simplified as

(8) $$\Delta x_{n}=\frac{\eta \omega \sigma_{ce}(uB-E_{R})}{x_{n}}t_{on}+\frac{\eta \omega \sigma_{ce}\tau }{x_{n}}\left[\left(uB-E_{R})\ln(1-\frac{E_{R}}{uB})+E_{R}\ln\frac{E_{R}}{uB}-E_{R}\ln(1-e^{-\frac{t_{on}}{\tau }}\right)\right]$$

Therefore, the side gap x at the position of h from the bottom of the electrode can be expressed as

(9) $$x=\sum_{n=0}^{\frac{h}{\nu T}}\Delta x_{n}+x_{0}$$

In Equation (9), the part of $x_{0}$ is machined by the bottom of electrode, which belongs to the primary electrochemical machining. The part of $\sum_{n=0}^{\frac{h}{\nu T}}\Delta x_{n}$ is machined by the side of electrode, which belongs to the secondary electrochemical machining. In the process of ECD, the primary electrochemical machining occupies a dominant position and has a great influence on the diameter of holes.

When other parameters are the same, at the side gap $x_{n}$, increasing the peak voltage u, the initial value of the side gap $x_{0}$ would increase and the increment of side gap $\Delta x_{n}$ would also increase, which would lead to the increase of the side gap x, then the diameter of the entrance would be increased.

In addition, the formation of the hole taper is mainly caused by the secondary electrochemical machining, which means the value of hole taper is decided by the part of $\sum_{n=0}^{\frac{h}{\nu T}}\Delta x_{n}$. Therefore, to machine holes with less taper, it is necessary to make the part of $\sum_{n=0}^{\frac{h}{\nu T}}\Delta x_{n}$ affected less by the depth h. Thus, to machine holes with less taper, the increment of the side gap $\Delta x_{n}$ should decrease to minimum as soon as possible.

### 2.3. Effects of Rotating Helical Electrode on Gap Flow Field

Helical electrode is a cylindrical electrode with micro helical groove on the surface; applying high-speed rotating helical electrode to electrochemical machining has a great effect on gap flow field. The structure of helical electrode is shown in Figure 3.

**Figure 4:**
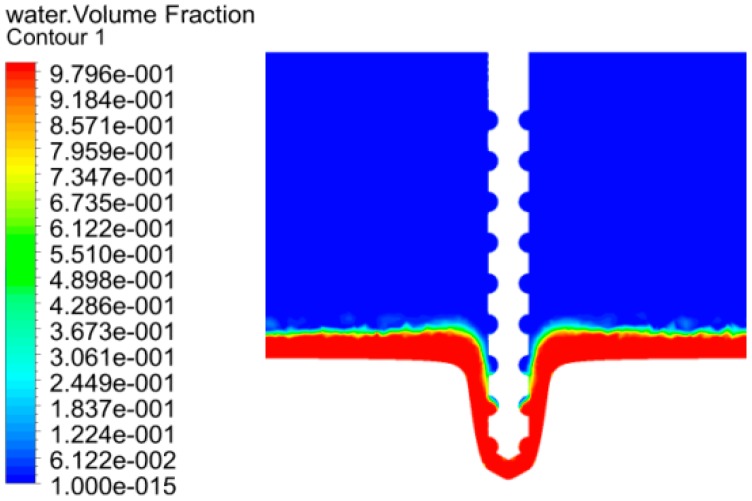
Distribution diagram of gas and liquid.

**Figure 5:**
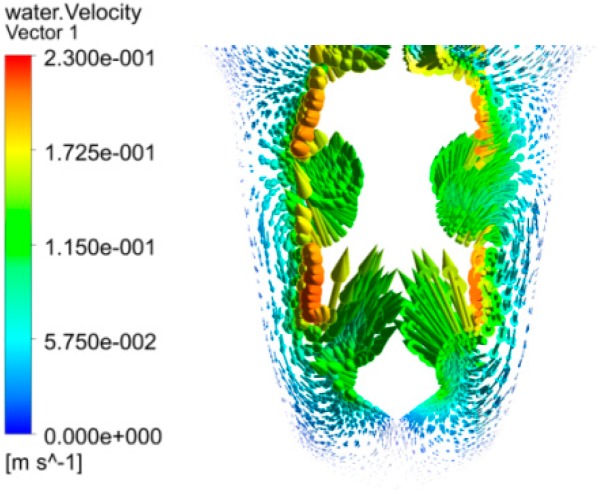
Velocity vector distribution diagram of electrolyte.

Using finite element analysis software, the flow field in the processing area was simulated. The distribution diagram of gas, liquid and the velocity vector distribution diagram of the electrolyte were obtained. The distribution gas and liquid are shown in Figure 4, in which the blue region means air and the red area means the electrolyte. It can be seen that there are some blue regions entering the entrance of the machining hole, which plays an insulating role on the side wall of the spiral electrode and reduces the conductivity $\sigma_{ce}$ of the side gap electrolyte. Figure 5 shows the velocity vector distribution diagram of the electrolyte, and it can be seen that the electrolyte flows in from the inner wall of the hole and flows out from the groove of the electrode to realize the renewal of the electrolyte, which contributes to take the electrolysis product off from the machining region to decrease the conductivity $\sigma_{di}$ of the electrolyte in the machining gap.

According to Equation (8), when the other parameters are constant, the conductivity $\sigma_{ce}$ of electrolyte in the side gap decreases, the side gap increment decreases and the side gap decreases, thus the diameter of entrances decreases and the machining accuracy increases. At the same time, the conductivity of the electrolyte in the side gap decreases, which would reduce the increment of the side gap, such that the increment could be soon enough to reduce the taper of the holes. On the other hand, the decrease of conductivity in the machining gap is less, to ensure the stabilization of the average machining gap; the feed rate would be bigger than applying the simple cylindrical electrode, which means the machining efficiency would be improved.

## 3. Results and Discussion

To verify the correctness of the theoretical model and analysis, a helical electrode was used as the tool cathode, and a GH4169 alloy plate was used as the workpiece. The experimental results were measured by Nikon optical microscope. Table 1 lists the common parameters applied in the experiments. The data represent average values, which were obtained by repeating the experiment at least three times.

### 3.1. Effect of Peak Voltage on the Diameter of Entrances 

**Figure 6:**
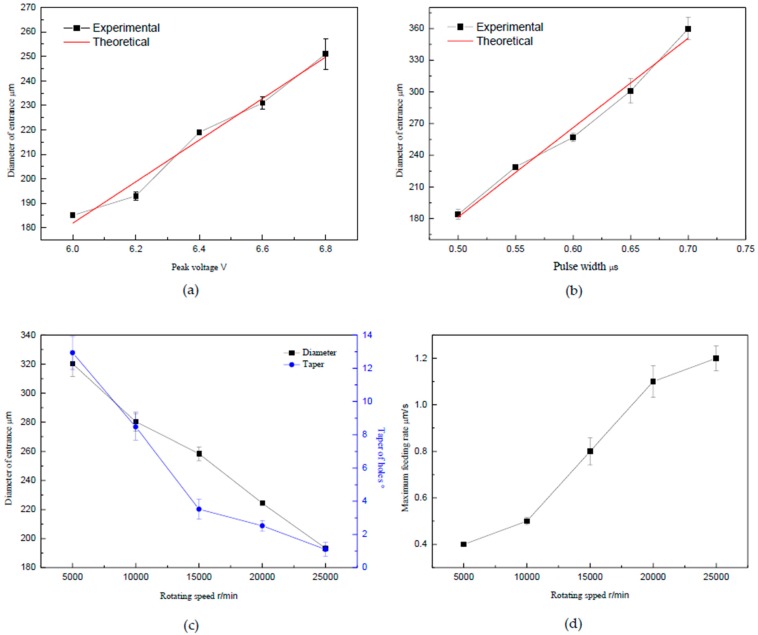
Curve of the diameter of entrance changing with the peak voltage: (**a**) curve of the diameter of entrance changing with the peak voltage; (**b**) curve of the diameter of entrance changing with the pulse width; (**c**) curve of the diameter of entrance and taper of holes changing with the rotating speed; and (**d**) curve of the maximum feed rate changing with the rotating speed.

Experiments were carried out to investigate the influence of peak voltage on machining accuracy. The applied parameters were as follows: the pulse cycle was 2.5 μs, the pulse width was 0.5 μs, the feed rate was 1.0 μm/s, and the rotating speed was 25,000 r/min. When the peak voltages were 6.0, 6.2, 6.4, 6.6 and 6.8 V, the curve of the diameter of entrances changed with the peak voltage, as shown in Figure 6a. The chosen range of data discussion is based on the experimental results shown in Figure S1.

As shown in Figure 6a, the diameter of entrance increased as the peak voltage increased, and the changing curve was approximately linear. According to Equation (5), since the rotating speed of the helical electrode was up to 25,000 r/min, the conductivity in the side wall was decreased greatly, thus side gap was mainly determined by the initial side gap $x_{0}$. The time constant τ was less than the pulse width 0.5 μs, thus the value of $\tau \left[\left(uB-E_{R})\ln(1-\frac{E_{R}}{uB})+E_{R}\ln\frac{E_{R}}{uB}-E_{R}\ln(1-e^{-\frac{t_{on}}{\tau }}\right)\right]$ was very small compared to the value of $(u-E_{R})t_{on}$. The initial side gap $x_{0}$ was mainly determined by the part of $\frac{\eta \omega \sigma (uB-E_{R})}{\nu T}t_{on}$, then the diameter of the entrance should theoretically increase linearly with the change of peak voltage. The changing trend of experimental results was consistent with the theoretical analysis, confirming the correctness of the theoretical analysis. 

### 3.2. Effect of Pulse Width on the Diameter of Entrance

Experiments were carried out to investigate the influence of pulse width on machining accuracy. The applied parameters were as follows: the peak voltage was 6.0 V, the pulse cycle was 2.5 μs, the feed rate was 1.0 μm/s, and the rotating speed was 25,000 r/min. Figure 6b shows the curve of the change of the diameter of entrance when the pulse widths were 0.5, 0.55, 0.6, 0.65 and 0.7 μs. The chosen range of data discussion is based on the experimental results shown in Figure S2.

As shown in Figure 6b, the diameter of entrance increased as the pulse width increased, and the changing curve was approximately linear. Based on the above analysis, the initial side gap $x_{0}$ was mainly determined by the part of $\frac{\eta \omega \sigma (uB-E_{R})}{\nu T}t_{on}$, thus the diameter of entrance should theoretically increase linearly with the changing of pulse width *t_on_*. The changing trend of experimental result was consistent with the theoretical analysis, confirming the correctness of the theoretical analysis.

### 3.3. Effect of Rotating Speed on the Diameter and Taper of Holes

To investigate the influence of rotating speed on machining accuracy and taper of holes, experiments were carried out. The applied parameters were as follows: the peak voltage was 6.0 V, the pulse cycle was 2.5 μs, the pulse width was 0.5 μs, and the feed rate was 0.4 μm/s. When the rotating speeds were 5000, 10,000, 15,000, 20,000 and 25,000 r/min, the curve of the diameter of entrance and taper of holes changed with the rotating speed, as shown in Figure 6c. The chosen range of data discussion is based on the simulation analysis and experimental results shown in Figures S3 and S4.

As shown in Figure 6c, with the increasing of rotating speed, the diameter of entrance decreased. The taper of holes decreased with the increase of rotating speed, while the changing rate was faster in the 5000–15,000 r/min range than in the 15,000–25,000 r/min range. With the increase of the electrode rotating speed, the gas content around the electrode increased; the gas core generated gradually extended and the end of the electrode was wrapped; this could prevent the effect of the secondary electrolysis efficiently; and the taper of the micro-holes was reduced or even eliminated. Thus, the gas core plays a good role in insulation.

### 3.4. Effect of Rotating Speed on the Maximum Feed Rate

Experiments were carried out to investigate the influence of rotating speed on the maximum feed rate. The applied parameters were as follows: the peak voltage was 6.0 V, the pulse cycle was 2.5 μs, and the pulse width was 0.5 μs. When the rotating speeds were 5000, 10,000, 15,000, 20,000 and 25,000 r/min, the curve of the maximum feed rate changed with the rotating speeds, as shown in Figure 6d.

As shown in Figure 6d, with the increase of the rotating speed, the maximum feed rate increased, which is shown in detail in Figure S5. The increase in electrode rotation speed raised the mean velocity magnitude of electrolyte in the machining gap. When the rotation speed of electrode achieved 25,000 r/min, the flow pattern at the near sidewall of gap fully rotated and the change of the overall flow patterns indicated that electrolyte was renovated quickly. Therefore, with the increase of rotational speed, the feed rate showed an upward trend.

**Figure 7:**
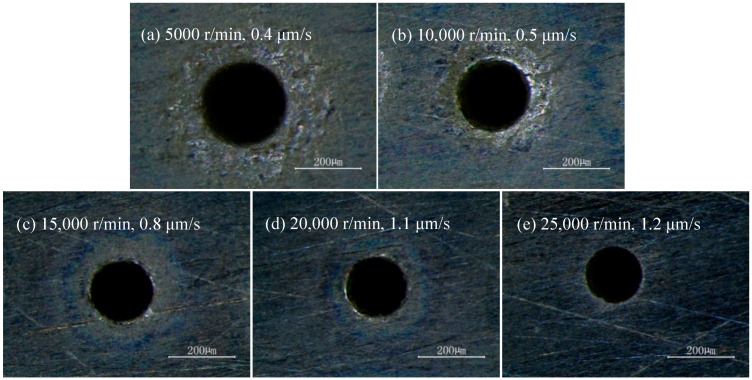
Results at the different rotating speed and the maximum feed rate.

The machining results at different rotating speeds and the corresponding maximum feed rates are shown in Figure 7. In Figure 7a,b, not only was the diameter of entrance large, but there were also large stray corrosions around the entrance. This was because, compared to high rotating speeds, the air entering the hole at the low rotating speed was insufficient to cover the surface of electrode. Thus, the conductivity was large in the side gap, which resulted in the large corrosion of the side wall and corrosion in the entrance near the surface. In Figure 7c–e, the rotating speed was greater than 15,000 r/min, which led to much air entering the hole, thus the conductivity decreasing more. Then, the corrosion rate at side wall was reduced, thus the diameter of entrance was smaller and there was basically no stray corrosion phenomenon around entrance.

According to the theoretical analysis and experimental results, applying the rotating helical electrode and ultra-short voltage pulse could improve machining accuracy, reduce taper of holes and improve machining efficiency. At the same time, the corresponding influence of various electrical parameters on the diameter of holes were researched theoretically and experimentally. Therefore, to get high-accuracy micro holes efficiently, the following optimized parameters should be used.

**Figure 8:**
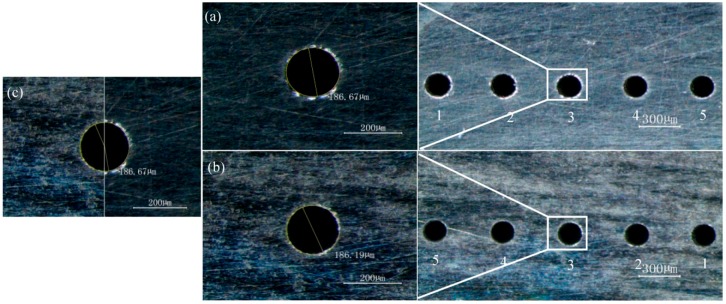
Micro array holes with no taper: (**a**) entrance; (**b**) exit; and (**c**) comparison of entrance and exit.

The machining results applying the optimized machining parameters are shown in Figure 8. The parameters were as follows: the peak voltage was 6.0 V, the pulse cycle was 2.5 μs, the pulse width was 0.5 μs, the rotating speed was 25,000 r/min, and the feed rate was 1.2 μm/s. The helical electrode with the diameter of 100 μm was used as tool electrode, while the GH4169 plate with the thickness of 500 μm was the workpiece. In Figure 8, the diameter of the exit and entrance are consistent. The measuring results by optical microscope show that the diameter of entrance was 186.67 μm while the diameter of exit was 186.19 μm, thus the taper of holes was almost zero. Relevant experiments were also carried out on GH4169 plates with larger thickness, as shown in Figure S6.

## 4. Conclusions

In this work, according to the analysis of theoretical model and experimental results, some conclusions can be obtained. According to the processing characteristics of ultra-short voltage pulse, the theoretical model of electrochemical drilling was established, and the influence of the peak voltage on the diameter of holes was analyzed. The high-speed rotating helical electrode could be used to reduce the diameter of holes, reduce the taper of holes, and increase the maximum feed rate in the process of electrochemical drilling. By applying the optimized parameters, taper free micro array holes with diameter of 186 μm were machined at the feed rate of 1.2 μm/s on the GH4169 plate of 500 μm thickness.

## Figures and Tables

**Table 1:** Common parameters applied in the experiments.

Parameters	Valve
Electrode diameter	100 μm
Thickness of alloy plate	500 μm
Concentration of electrolyte	5% NaNO_3_
Temperature of electrolyte	25 °C
Initial machining gap	5 μm

## Supplementary Materials

The following are available online at http://www.mdpi.com/2072-666X/10/1/28/s1, Figure S1: Machining results at different voltages, Figure S2: Machining results at different pulse widths, Figure S3: Distribution of water vapor at different rotating speed, Figure S4: Machining results at different rotating speed, Figure S5: The velocity vector graph of the flow field in micro machining gap, Figure S6: Deep and micro holes object.
