# Supplementary material for: Fabrication of Taper Free Micro-Holes Utilizing a Combined Rotating Helical Electrode and Short Voltage Pulse by ECM
Source: Micromachines (Basel). 2019 Jan 3;10(1):28. doi: 10.3390/mi10010028 (PMC6356685; doi:10.3390/mi10010028)
Supplement: Supplementary file 1 [file micromachines-10-00028-s001.pdf]

micromachines

Supplementary Materials: Fabrication of Taper Free Micro-Holes Utilizing a Combined Rotating Helical Electrode and Short Voltage Pulse by ECM

Yong Liu, Minghong Li, Jingran Niu, Shizhou Lu, Yong Jiang

The range of data discussion we choose is based on the experimental results. Taking the discussion of voltage range as an example, when the machining voltage exceeds 6.8 V, serious over cut and stray corrosion will occur in the machined holes as shown in Figure S1. We think that the case of machining voltage exceeding 6.8 V is not suitable for actual machining, which is not meaningful to discuss. So, we choose the range of 6.0 to 6.8 V to discuss. The choice of the discussion range is also applicable to pulse width. The following machining results are obtained under different voltages and pulse widths, as shown in Figure S1 and S2.

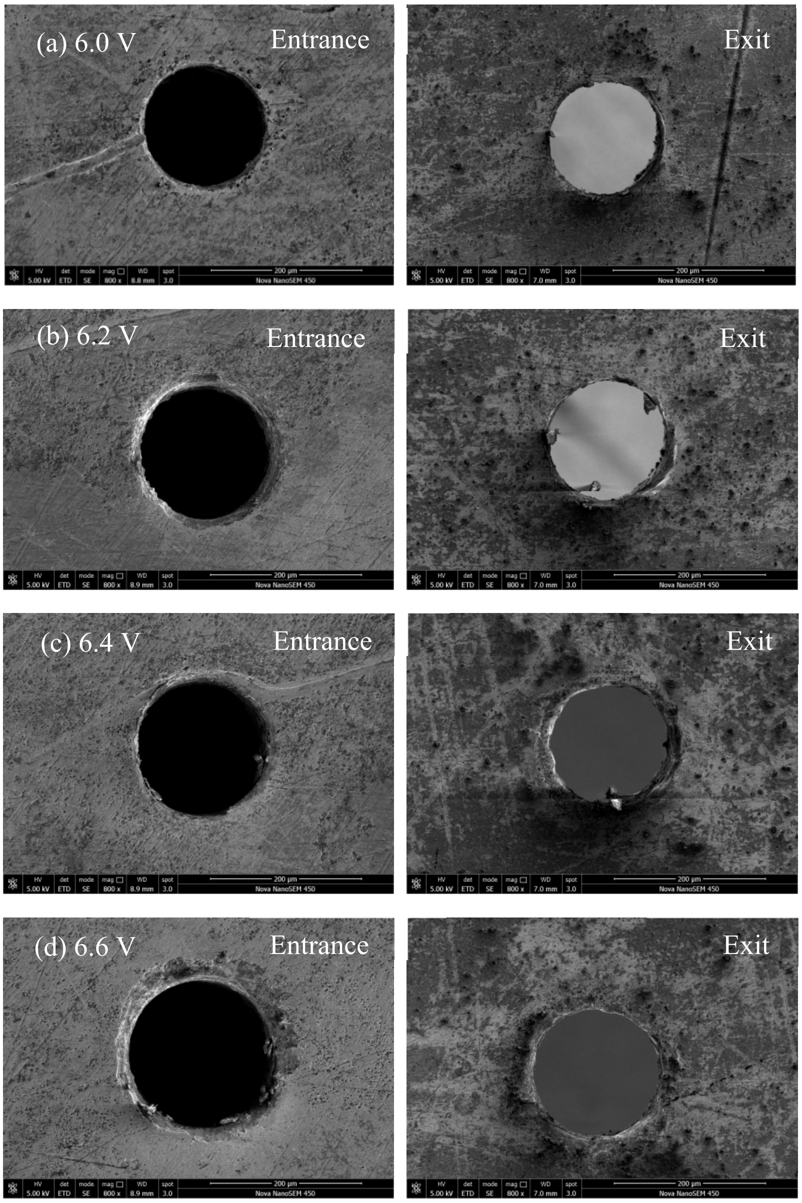

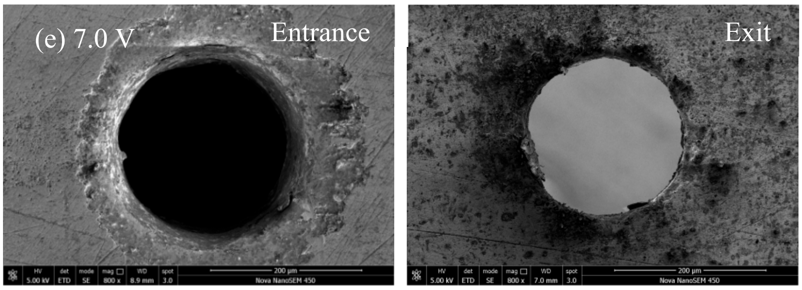

Figure S1. Machining results at different voltages.

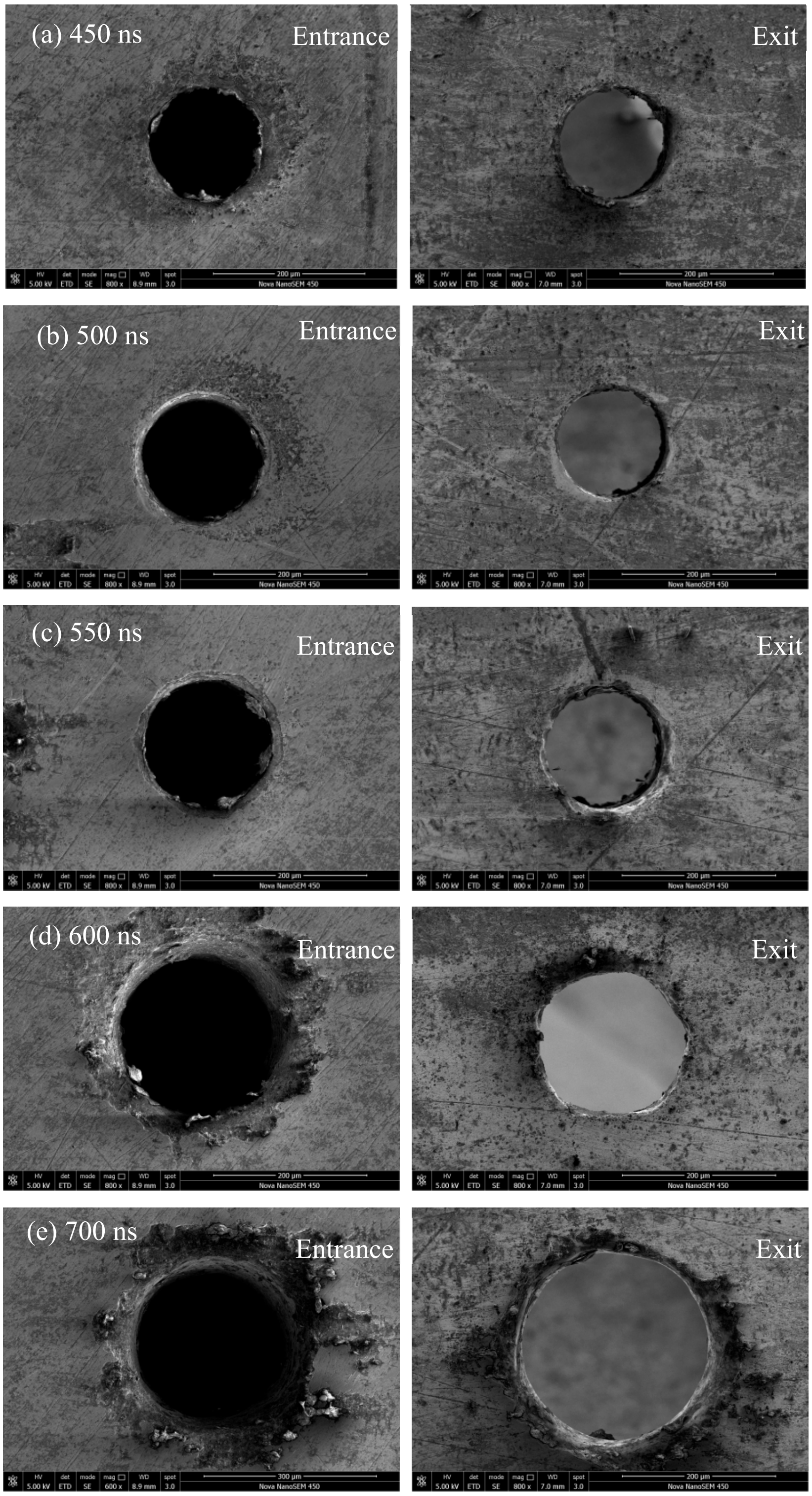

Figure S2. Machining results at different pulse widths.

The range of rotating speed discussion we choose is based on the analysis of water vapor distribution in Figure S3 and the experimental results as shown in Figure S4. Figure S3 is the water vapor distribution of two phase flow in micro machining gap. Figure S3a–f is the trend chart of water vapor distribution with different rotating speeds. From Figure S3, it is noticeable that when the spiral micro electrode rotates with high-speed, the flow around it will forms the vertical vortex flow of axial symmetry rapidly with the drive of the groove. With the increase of the electrode rotating speed, the gas content around the electrode also increase, the gas core generated is gradually extended and the end of the electrode is wrapped, which can prevent the effect of the secondary electrolysis efficiently, the taper of the micro-holes is reduced or even eliminated, so the gas core plays a good role in insulation. Due to space constraints, this analysis process in detail does not appear in the manuscript. In terms of experiments, when the rotating speed is low, serious over cut and stray corrosion will occur on the machined holes as shown in Figure S4a–e.

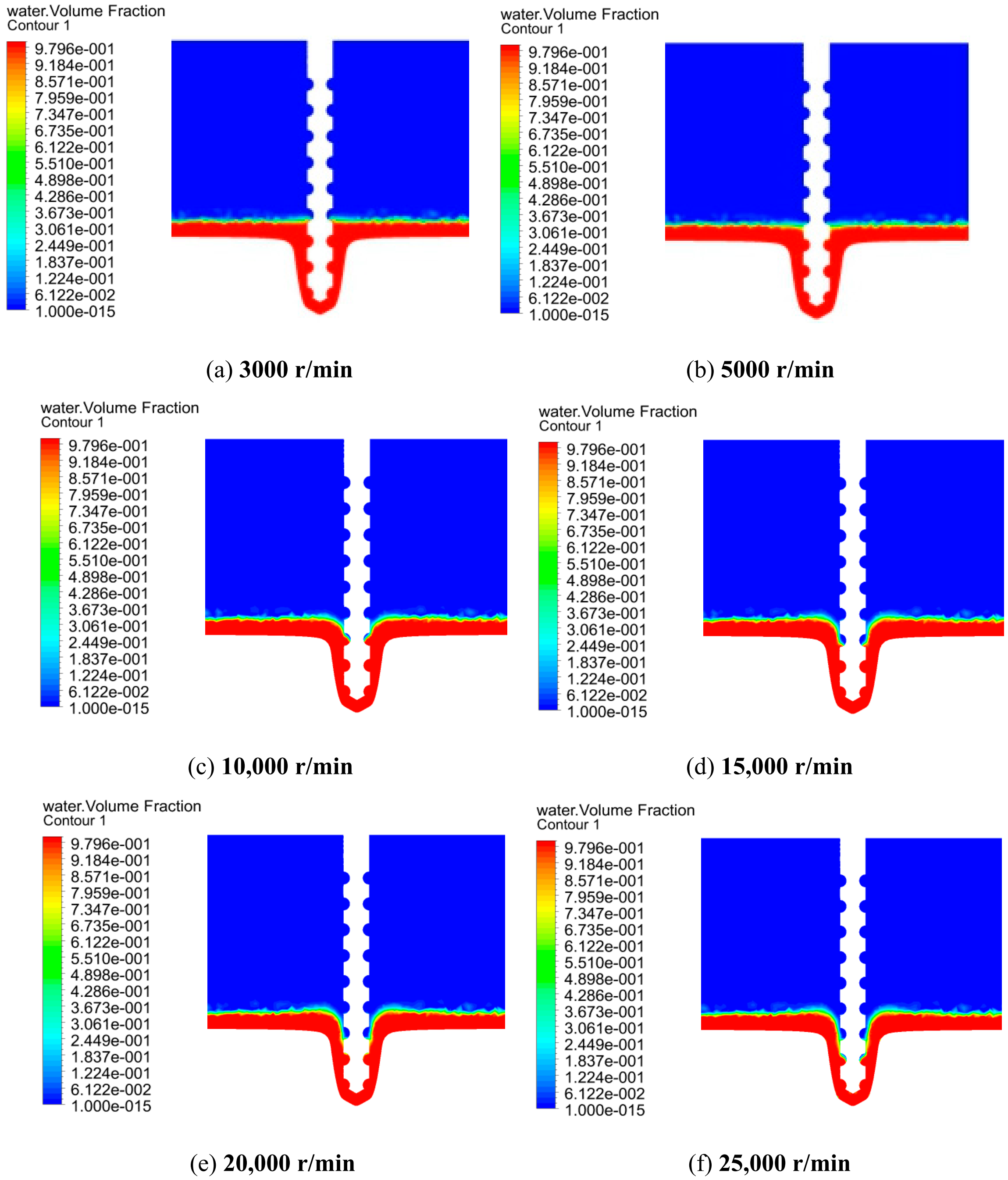

Figure S3. Distribution of water vapor at different rotating speed.

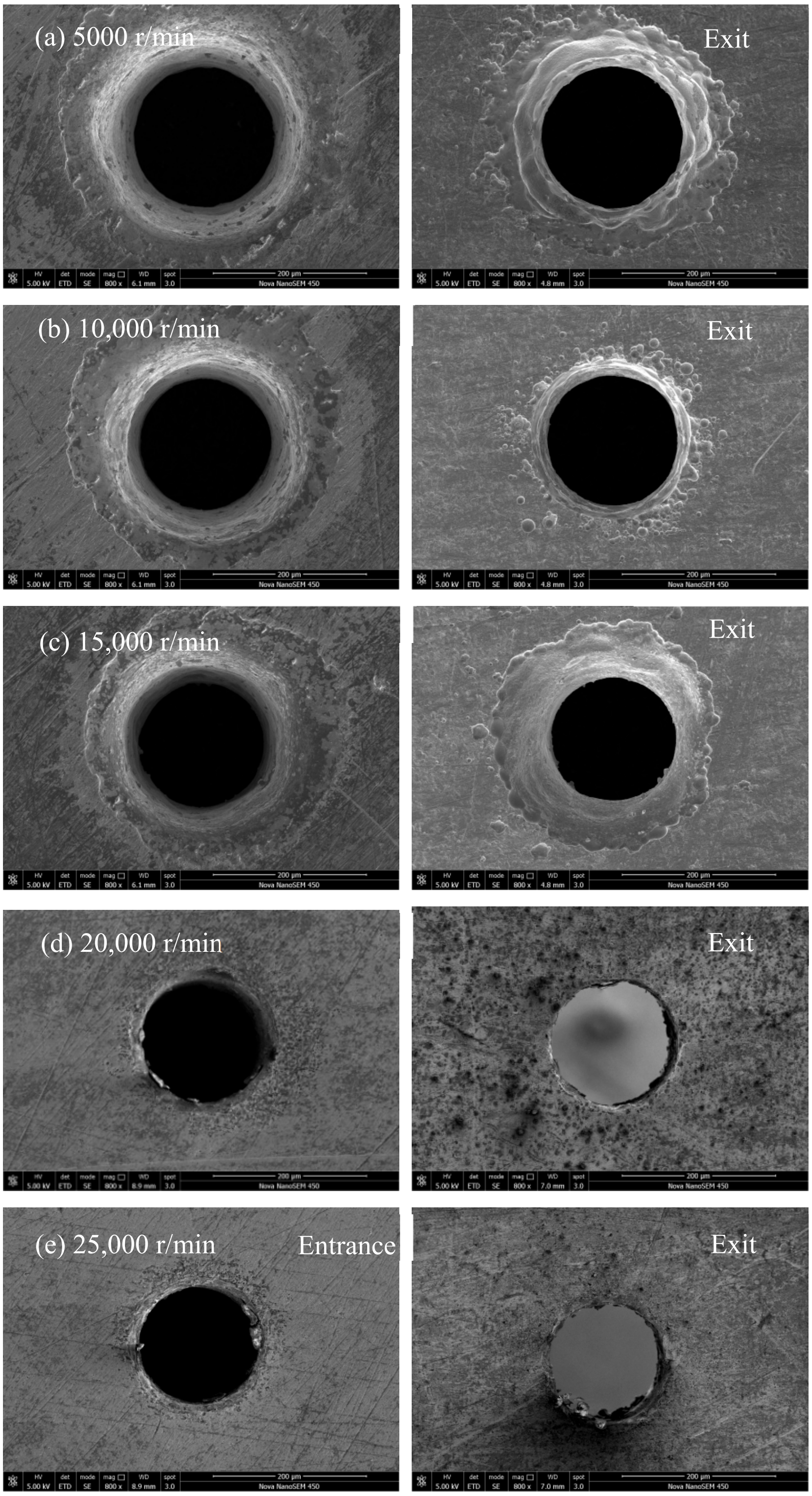

Figure S4. Machining results at different rotating speed.

From Figure S5, we can find that close to the side of the spiral electrode, the direction of the flow velocity is upward and along the wall, the direction is downward. It can be seen that the vertical vortex flow not only has the tangential velocity and radial velocity, but also has the upward axial velocity, so the reaction products can be removed. Meanwhile, because of the negative pressure generated by vortex wake, the fresh electrolyte outside the machining domains is inhaled into the machining domains along the side wall. Therefore, the electrolyte can be updated rapidly. The

increase in electrode rotation speed leads to raise mean velocity magnitude of electrolyte in the machining gap. when rotation speed of electrode achieve 25,000 r/min, the flow pattern at the near sidewall of gap fully rotation and the change of the overall flow patterns indicated that electrolyte was renovated fleetly. Therefore, with the increase of rotational speed, the feed rate shows an upward trend.

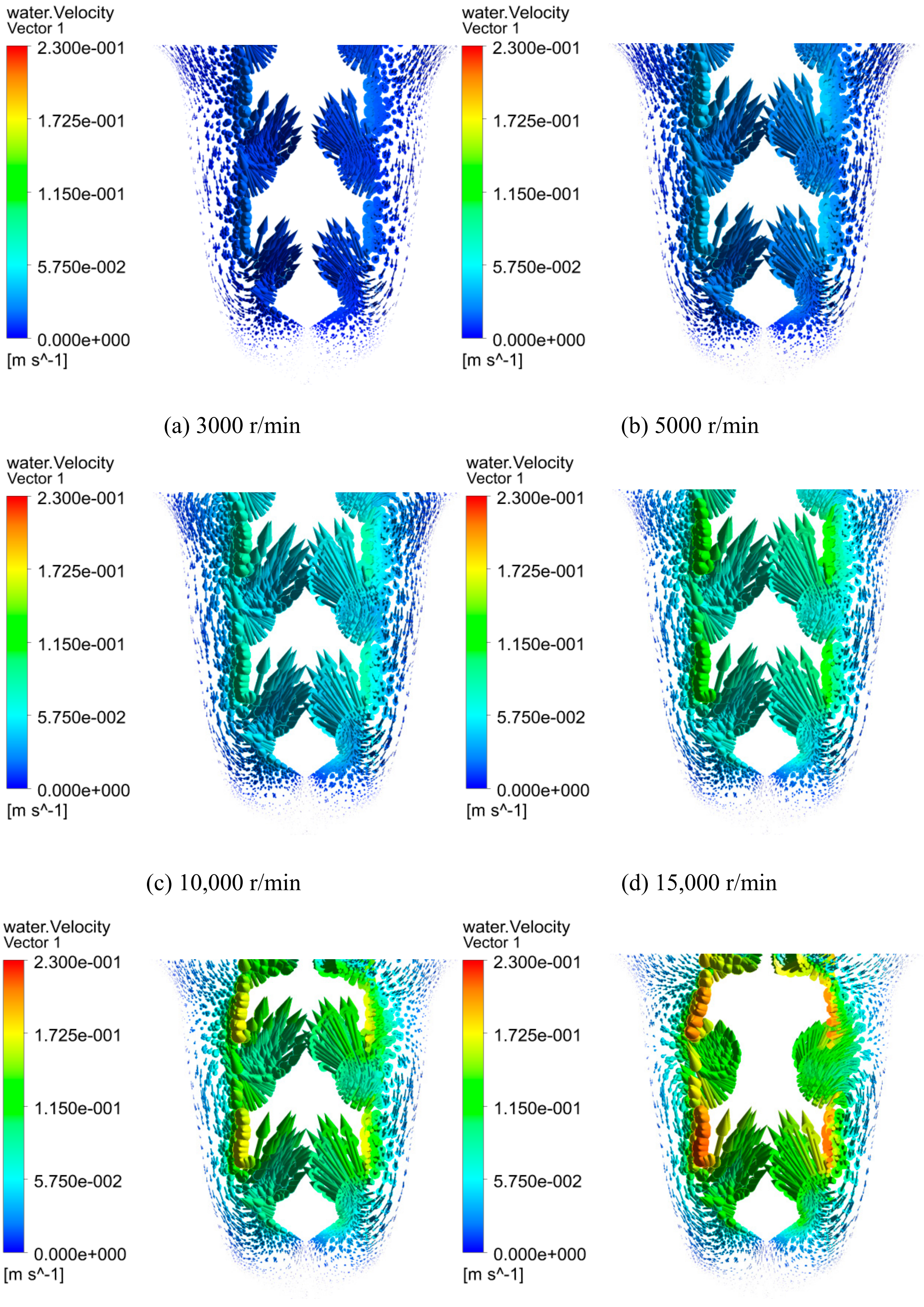

(a) 3000 r/min (b) 5000 r/min

(c) 10,000 r/min (d) 15,000 r/min

(e) 20,000 r/min (f) 25,000 r/min

Figure S5. The velocity vector graph of the flow field in micro machining gap.

Relevant experiments were also carried out on GH4169 plates with larger thickness as shown in Figure S6. Figure S6 show some graphs of deep micro holes of about 400 μm in diameter on a 4 mm thickness GH4169 plate, it can be seen that the wall of deep micro holes are all very steep, no taper, and with depth to diameter ratio of about 10:1.

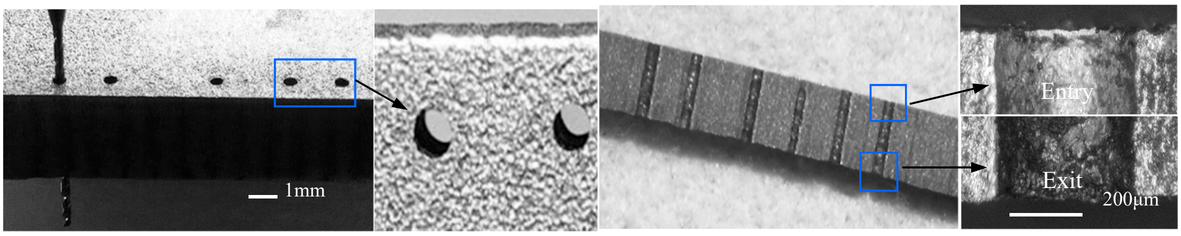

(a) micro electrode and holes (b) Enlarged view (c) Cutaway view (d) Enlarged view

Figure S6. Deep and micro holes object.
